# Commentary on “Pediatric Idiopathic Steroid-sensitive Nephrotic Syndrome Diagnosis and Therapy - Short version of the updated German Best Practice Guideline (S2e)”

**DOI:** 10.1007/s00467-021-05136-2

**Published:** 2021-06-05

**Authors:** Rasmus Ehren, Marcus R. Benz, Paul T. Brinkkötter, Jörg Dötsch, Wolfgang R. Eberl, Jutta Gellermann, Peter F. Hoyer, Isabelle Jordans, Clemens Kamrath, Markus J. Kemper, Kay Latta, Dominik Müller, Jun Oh, Burkhard Tönshoff, Stefanie Weber, Lutz T. Weber, on behalf of the German Society for Pediatric Nephrology

**Affiliations:** 1 Pediatric Nephrology, Children’s and Adolescents’ Hospital, Faculty of Medicine and University Hospital Cologne, University of Cologne, Cologne, Germany; 2 Department II of Internal Medicine and Center for Molecular Medicine Cologne (CMMC), Faculty of Medicine and University Hospital Cologne, University of Cologne, Cologne, Germany; 3 Cologne Cluster of Excellence on Cellular Stress Responses in Ageing-Associated Diseases (CECAD), Cologne, Germany; 4 Department of Pediatrics, Städtisches Klinikum Braunschweig, Braunschweig, Germany; 5 Pediatric Nephrology, Charité Children’s Hospital, Berlin, Germany; 6 Center for Children and Adolescents, Pediatric Clinic II, University of Duisburg-Essen, Essen, Germany; 7 Bundesverband Niere eV (German National Kidney-Patients Association), Mainz, Germany; 8 Division of Pediatric Endocrinology & Diabetology, Center of Child and Adolescent Medicine, Justus Liebig University, Giessen, Germany; 9 Department of Pediatrics, Asklepios Medical School, Hamburg, Germany; 10 Clementine Kinderhospital Frankfurt, Frankfurt, Germany; 11 Division of Pediatric Nephrology, Hepatology and Transplantation, University Medical Center Hamburg-Eppendorf, Hamburg, Germany; 12 Department of Pediatrics I, University Children’s Hospital, Heidelberg, Germany; 13 Department of Pediatrics II, University Children’s Hospital, Philipps-University Marburg, Marburg, Germany

Childhood idiopathic nephrotic syndrome is rare but represents the most common glomerular disease in the pediatric cohort. Glucocorticoid (steroid) treatment depicts the cornerstone of both treatment of initial episode as well as relapse. Sensitivity to steroids is of high importance for prognosis.

Pediatric nephrologists throughout the world would probably subscribe to these statements. Thereafter, however, management of the disease is dependent on individual experience and attitude rather than adherence to concerted recommendations. Consequently, more recent practice guidelines on management of pediatric nephrotic syndrome [1–3] do not necessarily conform in detail and leave room for interpretation. Variability of physicians’ adherence to these recommendations is potentially a result of a lack of evidence from randomized controlled studies [4–6], and treatment protocols in the real world may follow experiential (tacit) and empirical knowledge in decision-making rather than scientific evidence [5].

The short version of the updated German best practice guideline on pediatric idiopathic steroid-sensitive nephrotic syndrome (SSNS) published in this issue of *Pediatric Nephrology* [7] reflects the body of evidence until the end of 2019 and acknowledges the personal experience of a highly qualified guideline committee. This German guideline thus summarizes current knowledge and experience and gives best practice recommendations at a defined time point. However, since scientific activities on different aspects of therapy are ongoing and more recently published studies may have the potential to challenge certain statements of our guideline, we highly appreciate the invitation of the Editorial Board to comment on them, thereby continuing the steady discussion on best practice treatment.

In the following, we further address important issues raised by the high-quality review process that could not be implemented in the guideline report without undermining the strict creation process of the guideline. The subsequent main two topics also reflect the lack of distinct evidence and the corresponding variable approach driven by expertise.

- Standardized alternative treatment protocol of complicated courses of SSNS, namely frequently relapsing nephrotic syndrome (FRNS) and steroid-dependent nephrotic syndrome (SDNS)

In clinical practice, we are often confronted with the wish for a standardized alternative treatment protocol for complicated courses of SSNS. Likewise, this wish appeared in the review process of our guideline that lists alternative immunosuppressive drugs to avoid toxicity of repeated or long-term courses of steroid therapy and grades the corresponding statements according to the rating guideline used by KDIGO [3]. Recommendations on a hierarchical order, however, cannot be given due to a lack of randomized controlled head-to-head comparative studies. We strongly believe that children suffering from complicated courses of nephrotic syndrome should be cared for by specialized institutions with a focus on pediatric nephrology, since decision-making on therapeutic management is highly individual. Decision-making is dependent on factors concerning the health care provider such as experiential and empirical knowledge, personal preferences or recent patient experiences as well as patient characteristics such as family preferences, ethnicity, meaning of potential drug toxicity to the individual patient, and individual experiences with response and adherence to drug treatments [5]. Our guideline recommends mycophenolate mofetil (MMF) or calcineurin inhibitors (CNIs) to begin steroid-sparing alternative treatment when indicated and reserves rituximab for non-responders and those with explicit side effects. More generally, pediatric nephrologists might indicate MMF as their first choice based on a favorable side effect profile compared to CNIs with nephrotoxicity being the most important limitation [4]. The randomized controlled trial (RCT) performed by the German Society for Pediatric Nephrology (GPN) showed higher efficacy of cyclosporine (CsA) versus MMF in patients with FRNS [8]. This crossover study indicated a potential carry-over effect of CsA with more relapses per patient per year with MMF than with CsA during the first, but not during the second year. Superior efficacy of CsA disappeared when the group of patients with adequate exposure to mycophenolic acid (MPA) (MPA area under the curve > 50 mg × h/L) was analyzed post hoc, indicating the importance of therapeutic drug monitoring for MMF. Combination of drugs may be more effective than monotherapy and can be an option in difficult-to-treat patients [9, 10]. Our guideline reserves rituximab for patients that fail under therapy with CNIs or MMF or experience severe side effects under these medications. Future study results may challenge this assessment. Ravani et al. showed non-inferiority of a single dose of rituximab toward steroid therapy in children with SDNS in terms of percentage change of proteinuria in follow-up [11]. Of note, patients with prior CNI therapy had been excluded from study participation. Both patient groups were weaned off steroids shortly after study entry. It was therefore no surprise that patients in the rituximab group did better than those in the control group who were divested exactly the therapy that had kept them in remission beforehand. In addition, patient number was small (n = 30) and comprised only patients being responsive to rather low doses of maintenance steroids. The most interesting finding of this trial is reflected by long-term remission of 8/15 patients after 4 years of single rituximab application. This number is distinctly higher than that reported in children with multidrug dependence [12] and challenges the finding in Japanese patients, who experienced a high rate of relapses within the first year after a single dose of rituximab [13]. Another trial from the same group compared a rituximab biosimilar versus low-dose MMF in children with SDNS [14]. This trial was stopped prematurely due to high rates of relapse in the MMF arm. The reason to choose substandard dosing of MMF in this study remains unexplained. Therapeutic drug monitoring of MMF had not been performed. It remains unclear whether the risk of relapse in the MMF arm had been a consequence of insufficient exposure to mycophenolic acid.

We do not recommend performing a kidney biopsy before starting alternative steroid-sparing maintenance therapy in SSNS unless presentation as nephritic syndrome or suspected systemic disease. The nephrotoxic burden of CNIs is occasionally mentioned as an argument for an initial finding. We do not share this attitude, taking the potential complications of the procedure into account [15, 16]. Figure 1 shows a potential treatment algorithm for FRNS and SDNS in the pediatric cohort.

**Fig. 1:**
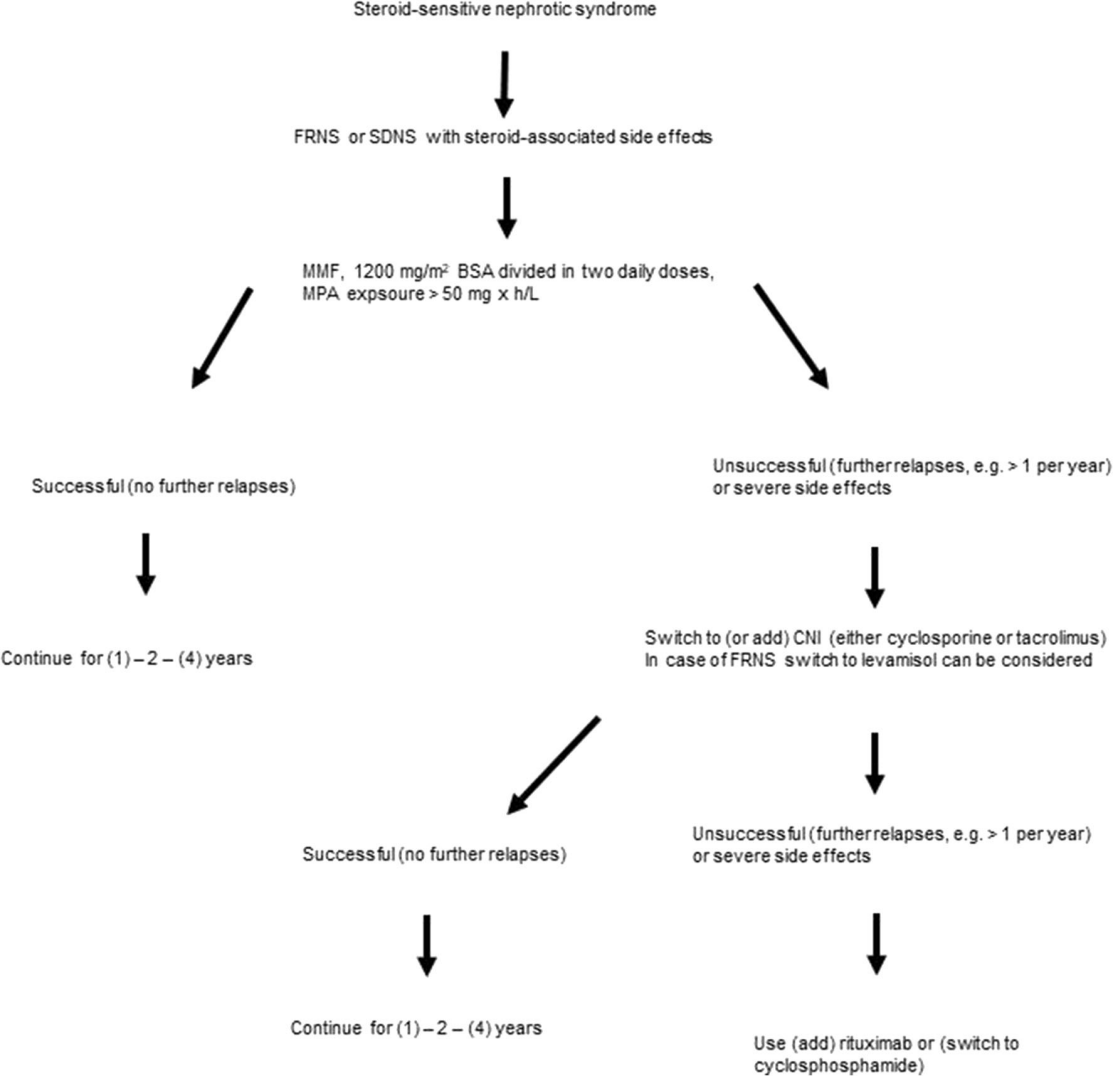
Potential algorithm for alternative immunosuppressive treatment of FRNS and SDNS. Abbreviations: BSA, body surface area; CNI, calcineurin inhibitor; FRNS, frequently relapsing nephrotic syndrome; MMF, mycophenolate mofetil; MPA, mycophenolic acid; SDNS, steroid-dependent nephrotic syndrome

We are well aware of variable opinions on when to start alternative treatment in the course of the disease. Physicians may not wait for steroid-associated side effects but would prefer to consider cumulative steroid dose and the corresponding risk of side effects for intervention. Again, decision-making will be influenced by patients’ as well as physicians’ concerns as described above. The same holds true for the duration of alternative treatment. Its optimal duration is currently unknown. Very early reports describe an omission attempt after 6 months of therapy [17, 18]. Newer recommendations depict a treatment duration of at least 12 months for CNIs [3] and several years for MMF [9]. Others even abstain from recommending any timeframe [19]. A recent survey among Dutch pediatricians and pediatric nephrologists revealed a majority going for 12 to 24 months duration of maintenance therapy for FRNS and SDNS [4]. High risk of relapse after cessation of alternative treatment is reported uniformly. Restart of alternative treatment, however, results in stable remission [20]. Therefore, tentative discontinuation of alternative treatment after stable remission for 1–4 years does not come with unacceptable future risk of numerous relapses, but may be helpful to reduce potential side effects evoked by long-term use.

- Steroids for initial therapy and treatment of relapse

The treatment with prednisone in the “lowest dose required” to maintain remission is common in many countries. The British Association of Paediatric Nephrology and the Indian Pediatric Nephrology Group recommend long-term low-dose administration of prednisone in FRNS to prevent relapse. The KDIGO guidelines also suggest (level of evidence 2D) to use the lowest possible steroid dose to maintain remission and, if an alternating dosing regimen is not successful, to switch to daily administration of the same dose [3]. Since the evidence is low (no RCTs) and the empirically based recommendations are not proven, this approach can only be considered in individual cases. A recent RCT from India showed that daily administration of prednisolone was more effective than alternate-day prednisolone in a similar cumulative dose in preventing relapses during a 12-month observational period [21]. Patients in this study had a rather mild course of the disease. Relatively short follow-up potentially resulted in underreporting of long-term side effects such as osteoporosis and behavioral effects. Further confirmation of study results is therefore necessary, also considering potential impact on the hypothalamus-pituitary-adrenal axis. Nevertheless, this study speaks for low-dose daily rather than alternate-day prednisolone to maintain remission in FRNS and SDNS.

Reducing steroid toxicity is a primary issue in current management of childhood nephrotic syndrome. While treatment of the initial episode is fairly well established with a number of RCTs that did not show any benefit of longer duration of initial steroid therapy [22–25], duration and dose in relapse treatment are more variable. The PROPINE (Prospective Randomized study to Optimize Prednisone therapy for relapses of Idiopathic NEphrotic syndrome in children) study from Italy compared a short treatment arm (36 days) versus a long treatment arm (72 days) for relapse from day 5 after remission using the same cumulative dose of prednisone [26]. The relapse rate was not different between the groups at 6 and 12 months. No difference in adverse events was noted between the groups. This study shows that it is not reasonable to prolong treatment schedules for relapse of nephrotic syndrome. The strength of this study was a secondary crossover design that allowed comparison of both treatment arms in the same patient and revealed similar results. Importantly, the primary study was slightly underpowered. A different approach was chosen in the study by Kainth et al. from India, who evaluated the efficacy of prednisolone as a “short regimen” (40 mg/m^2^ on alternate days for 2 weeks) compared with “standard regimen” (40 mg/m^2^ on alternate days for 4 weeks) for children aged 1–16 years who achieved remission of a relapse in a single-center, open-label, randomized controlled non-inferiority trial [27]. Even though non-inferiority could not be established, the short steroid treatment for relapse resulted in a similar proportion of patients developing frequent relapses or steroid dependence and resulted in a significantly lower cumulative steroid dose, yet steroid-related adverse events were similar in both groups. The ongoing RESTERN study (double-blind, randomized, placebo-controlled, non-inferiority intervention study to REduce STEroids in Relapsing Nephrotic syndrome; EudraCT 2016-002430-76) addresses the same topic, also in a non-inferiority design [28]. In the future, the results of the two latter studies may have the potential to change the recommendation on the duration of alternate-day steroid in treatment of relapses of SSNS in children toward a reduction to 2 weeks after remission has been achieved.

Very recently, Sheikh et al. published the results of an RCT comparing prednisolone 1 mg/kg/day (low dose) and 2 mg/kg/day (standard dose) as relapse treatment in a pediatric cohort with SSNS [29]. This trial could show non-inferiority of the low-dose arm regarding time to remission. After achieving remission, both study arms were treated in the same manner with prednisolone 1.5 mg/kg on alternating days for another 4 weeks. There was a tendency toward more relapses as well as of being diagnosed with FRNS or SDNS more often in the low-dose arm over the follow-up of 1 year. This might explain that there was no difference in the cumulative steroid dose in the entire follow-up between the groups. This may raise the question of which end points should be used when the study target is reduced exposure to steroids. Of note, younger children below the age of 4 to 6 years may have an advantage of an extended course of steroids in the first flare [24, 25, 30]. There are currently two ongoing clinical trials of 3 versus 6 months: a Chinese trial in children under 6 years (NCT04536181) and an Indian trial in children under 4 years (CTRI/2015/06/005939) to challenge the conclusion of the recent Cochrane update [31] against extended initial steroid duration [32]. Results of these trials may open the door for a more individual age-dependent treatment approach.

International [33] and national [4] surveys depict considerable differences in the maximal daily dose of steroids ranging from 60 to 80 to 100 mg. With respect to the general steroid minimizing approach, a maximum daily dose is to be debated. There is high inter-individual variability in the response to steroid treatment [34]. Pharmacogenetics can have an influence on both pharmacokinetics and pharmacodynamics of steroids in the individual patient. Large-scale studies are needed to draw final conclusions on individual steroid response. Up to the present time, there is personal experience of a higher than 60 mg daily steroid requirement in individual patients with body surface area beyond 1.5 m^2^.

This commentary would not be complete without pointing to a number of ongoing trials sharing the potential to influence future updates of our guideline:

- Mycophenolate mofetil as maintenance therapy after rituximab treatment for childhood-onset, complicated, frequently-relapsing nephrotic syndrome or steroid-dependent nephrotic syndrome: a multicenter double-blind, randomized, placebo-controlled trial (JSKDC07);
- Compare Efficacy and Safety of Repeated Courses of Rituximab to That of Maintenance Mycophenolate Mofetil Following Single Course of Rituximab Among Children With Steroid Dependent Nephrotic Syndrome (RITURNS II) (NCT03899103);
- Prevention of relapse with levamisole as adjuvant therapy to corticosteroids in children with first episode of idiopathic nephrotic syndrome (LEARNS) (EudraCT: 2017-001025-41);
- Efficiency of Levamisole for Maintaining Remission After the First Flare of Steroid Sensitive Nephrotic Syndrome in Children (NEPHROVIR3) (NCT02818738);
- Study of Tacrolimus vs Mycophenolate Mofetil in Pediatric Patients With Nephrotic Syndrome (STAMP) (NCT04048161).

A final word on management of complications: children with idiopathic nephrotic syndrome may suffer from massive edema with ascites and pleural effusions with hypovolemia and normal kidney function. In these children, concentrated albumin can be given together with furosemide [35]. Our guideline gives an infusion time for albumin of 1 to 2 h. Historical literature reports infusion times of 1 h or less [36, 37]. However, tolerability of albumin infusions is better with prolonged infusion times. This might be especially true for patients in whom extra caution is indicated, e.g., those with oliguria or significantly impaired kidney function. Suggested infusion times differ between 1 and 4 h, dependent on the amount of albumin infused. Very recent literature suggests 3 to 4 h infusion time for an amount of 1 g/kg BW of 20% albumin [38, 39]. We acknowledge this information and will consider it in the next update of our guideline.

In conclusion, management of childhood nephrotic syndrome is subject to constant change. Clinical practice recommendations, therefore, need regular updates to incorporate topical knowledge. Further efforts are underway dealing with the important topics of steroid-sparing and alternative immunosuppressive treatment in complicated courses.

## References

[1] Pasini A, Benetti E, Conti G, Ghio L, Lepore M, Massella L, Molino D, Peruzzi L, Emma F, Fede C, Trivelli A, Maringhini S, Materassi M, Messina G, Montini G, Murer L, Pecoraro C, Pennesi M (2017). The Italian Society for Pediatric Nephrology (SINePe) consensus document on the management of nephrotic syndrome in children: part I - diagnosis and treatment of the first episode and the first relapse. Ital J Pediatr.

[2] Samuel S, Bitzan M, Zappitelli M, Dart A, Mammen C, Pinsk M, Cybulsky AV, Walsh M, Knoll G, Hladunewich M, Bargman J, Reich H, Humar A, Muirhead N (2014). Canadian Society of Nephrology commentary on the 2012 KDIGO clinical practice guideline for glomerulonephritis: management of nephrotic syndrome in children. Am J Kidney Dis.

[3] (2012) Chapter 3: steroid-sensitive nephrotic syndrome in children. Kidney Int Suppl 2:163-171 10.1038/kisup.2012.16 PMC4089737 25028636

[4] Schijvens AM, van der Weerd L, van Wijk JAE, Bouts AHM, Keijzer-Veen MG, Dorresteijn EM, Schreuder MF (2021) Practice variations in the management of childhood nephrotic syndrome in the Netherlands. Eur J Pediatr. 10.1007/s00431-021-03958-8 10.1007/s00431-021-03958-8 PMC8105198 33532891

[5] Samuel SM, Flynn R, Zappitelli M, Dart A, Parekh R, Pinsk M, Mammen C, Wade A, Scott SD; Canadian Childhood Nephrotic Syndrome Project Team* (2017) Factors influencing practice variation in the management of nephrotic syndrome: a qualitative study of pediatric nephrology care providers. CMAJ Open 5:E424-E430 10.9778/cmajo.20160078 PMC5498309 28592406

[6] Pasini A, Aceto G, Ammenti A, Ardissino G, Azzolina V, Bettinelli A, Cama E, Cantatore S, Crisafi A, Conti G, D’Agostino M, Dozza A, Edefonti A, Fede C, Groppali E, Gualeni C, Lavacchini A, Lepore M, Maringhini S, Mariotti P, Materassi M, Mencarelli F, Messina G, Negri A, Piepoli M, Ravaglia F, Simoni A, Spagnoletta L, Montini G, NefroKid Study Group (2015). Best practice guidelines for idiopathic nephrotic syndrome: recommendations versus reality. Pediatr Nephrol.

[7] Ehren R, Benz MR, Brinkkötter PT, Dötsch J, Eberl WR, Gellermann J, Hoyer PF, Jordans I, Kamrath C, Kemper MJ, Latta K, Müller D, Oh J, Tönshoff B, Weber S, Weber LT (2021) Pediatric idiopathic steroid-sensitive nephrotic syndrome: diagnosis and therapy - short version of the updated German best practice guideline (S2e) - AWMF register no. 166-001, 6/2020. Pediatr Nephrol. 10.1007/s00467-021-05135-3 10.1007/s00467-021-05135-3 PMC8445869 34091756

[8] Gellermann J, Weber L, Pape L, Tönshoff B, Hoyer P, Querfeld U, Gesellschaft für Pädiatrische Nephrologie (GPN) (2013). Mycophenolate mofetil versus cyclosporin A in children with frequently relapsing nephrotic syndrome. J Am Soc Nephrol.

[9] Querfeld U, Weber LT (2018). Mycophenolate mofetil for sustained remission in nephrotic syndrome. Pediatr Nephrol.

[10] Wu B, Mao J, Shen H, Fu H, Wang J, Liu A, Gu W, Shu Q, Du L (2015). Triple immunosuppressive therapy in steroid-resistant nephrotic syndrome children with tacrolimus resistance or tacrolimus sensitivity but frequently relapsing. Nephrology (Carlton).

[11] Ravani P, Lugani F, Pisani I, Bodria M, Piaggio G, Bartolomeo D, Prunotto M, Ghiggeri GM (2020). Rituximab for very low dose steroid-dependent nephrotic syndrome in children: a randomized controlled study. Pediatr Nephrol.

[12] Chan EY-H, Webb H, Yu E, Ghiggeri GM, Kemper MJ, Ma AL-T, Yamamura T, Sinha A, Bagga A, Hogan J, Dossier C, Vivarelli M, Liu ID, Kamei K, Ishikura K, Saini P, Tullus K (2020). Both the rituximab dose and maintenance immunosuppression in steroid-dependent/frequently-relapsing nephrotic syndrome have important effects on outcomes. Kidney Int.

[13] Kamei K, Ito S, Nozu K, Fujinaga S, Nakayama M, Sako M, Saito M, Yoneko M, Iijima K (2009). Single dose of rituximab for refractory steroid-dependent nephrotic syndrome in children. Pediatr Nephrol.

[14] Ravani P, Lugani F, Drovandi S, Caridi G, Angeletti A, Ghiggeri GM (2021) Rituximab vs low-dose mycophenolate mofetil in recurrence of steroid-dependent nephrotic syndrome in children and young adults: a randomized clinical trial. JAMA Pediatr. 10.1001/jamapediatrics.2020.6150 10.1001/jamapediatrics.2020.6150 PMC7900932 33616641

[15] Feneberg R, Schaefer F, Zieger B, Waldherr R, Mehls O, Schärer K (1998). Percutaneous renal biopsy in children: a 27-year experience. Nephron.

[16] Sinha MD, Lewis MA, Bradbury MG, Webb NJA (2006). Percutaneous real-time ultrasound-guided renal biopsy by automated biopsy gun in children: safety and complications. J Nephrol.

[17] Brodehl J, Brandis M, Helmchen U, Hoyer PF, Burghard R, Ehrich JH, Zimmerhackl RB, Klein W, Wonigeit K (1998). Cyclosporin A treatment in children with minimal change nephrotic syndrome and focal segmental glomerulosclerosis. Klin Wochenschr.

[18] Hoyer PF, Brodehl J, Ehrich JH, Offner G (1991). Practical aspects in the use of cyclosporin in paediatric nephrology. Pediatr Nephrol.

[19] Larkins N, Kim S, Craig J, Hodson E (2016). Steroid-sensitive nephrotic syndrome: an evidence-based update of immunosuppressive treatment in children. Arch Dis Child.

[20] Vogd J, Hackl A, Habbig S, Lechner F, Akarkach A, Weber LT, Ehren R (2019). Discontinuation of maintenance therapy in frequently relapsing nephrotic syndrome. Clin Nephrol.

[21] Yadav M, Sinha A, Khandelwal P, Hari P, Bagga A (2019). Efficacy of low-dose daily versus alternate-day prednisolone in frequently relapsing nephrotic syndrome: an open-label randomized controlled trial. Pediatr Nephrol.

[22] Teeninga N, Kist-van Holthe JE, van Rijswijk N, de Mos NI, Hop WCJ, Wetzels JFM, van der Heijden AJ, Nauta J (2013). Extending prednisolone treatment does not reduce relapses in childhood nephrotic syndrome. J Am Soc Nephrol.

[23] Yoshikawa N, Nakanishi K, Sako M, Oba MS, Mori R, Ota E, Ishikura K, Hataya H, Honda M, Ito S, Shima Y, Kaito H, Nozu K, Nakamura H, Igarashi T, Ohashi Y, Iijima K, Japanese Study Group of Kidney Disease in Children (2015). A multicenter randomized trial indicates initial prednisolone treatment for childhood nephrotic syndrome for two months is not inferior to six-month treatment. Kidney Int.

[24] Sinha A, Saha A, Kumar M, Sharma S, Afzal K, Mehta A, Kalaivani M, Hari P, Bagga A (2015). Extending initial prednisolone treatment in a randomized control trial from 3 to 6 months did not significantly influence the course of illness in children with steroid-sensitive nephrotic syndrome. Kidney Int.

[25] NJA W, Woolley RL, Lambe T, Frew E, Brettell EA, Barsoum EN, Trompeter RS, Cummins C, Deeks JJ, Wheatley K, Ives NJ, PREDNOS Collaborative Group (2019). Long term tapering versus standard prednisolone treatment for first episode of childhood nephrotic syndrome: phase III randomised controlled trial and economic evaluation. BMJ.

[26] Gargiulo A, Massella L, Ruggiero B, Ravà L, Ciofi Degli Atti M, Materassi M, Lugani F, Benetti E, Morello W, Molino D, Mattozzi F, Pennesi M, Maringhini S, Pasini A, Gianoglio B, Pecoraro C, Montini G, Murer L, Ghiggeri GM, Romagnani P, Vivarelli M, Emma F (2021). Results of the PROPINE randomized controlled study suggest tapering of prednisone treatment for relapses of steroid sensitive nephrotic syndrome is not necessary in children. Kidney Int.

[27] Kainth D, Hari P, Sinha A, Pandey S, Bagga A (2021). Short-duration prednisolone in children with nephrotic syndrome relapse: a noninferiority randomized controlled trial. Clin J Am Soc Nephrol.

[28] Schijvens AM, Dorresteijn EM, Roeleveld N, Ter Heine R, van Wijk JAE, Bouts AHM, Keijzer-Veen MG, van de Kar NCAJ, van den Heuvel LPWJ, Schreuder MF (2017). REducing STEroids in Relapsing Nephrotic syndrome: the RESTERN study-protocol of a national, double-blind, randomised, placebo-controlled, non-inferiority intervention study. BMJ Open.

[29] Sheikh S, Mishra K, Kumar M (2021) Low-dose versus conventional-dose prednisolone for nephrotic syndrome relapses: a randomized controlled non-inferiority trial. Pediatr Nephrol. 10.1007/s00467-021-05048-1 10.1007/s00467-021-05048-1 33861375

[30] Hiraoka M, Tsukahara H, Matsubara K, Tsurusawa M, Takeda N, Haruki S, Hayashi S, Ohta K, Momoi T, Ohshima Y, Suganuma N, Mayumi M, West Japan Cooperative Study Group of Kidney Disease in Children (2003). A randomized study of two long-course prednisolone regimens for nephrotic syndrome in children. Am J Kidney Dis.

[31] Hahn D, Samuel SM, Willis NS, Craig JC, Hodson EM (2020) Corticosteroid therapy for nephrotic syndrome in children. Cochrane Database Syst Rev. https://www.cochranelibrary.com/cdsr/doi/10.1002/14651858.CD001533.pub6/full. Accessed 8 December 2020

[32] Christian MT, Maxted AP (2021) Optimizing the corticosteroid dose in steroid-sensitive nephrotic syndrome. Pediatr Nephrol. 10.1007/s00467-021-04985-1 10.1007/s00467-021-04985-1 PMC7896825 33611671

[33] Deschênes G, Vivarelli M, Peruzzi L, ESPN Working Group on Idiopathic Nephrotic Syndrome (2017). Variability of diagnostic criteria and treatment of idiopathic nephrotic syndrome across European countries. Eur J Pediatr.

[34] Schijvens AM, ter Heine R, de Wildt SN, Schreuder MF (2019). Pharmacology and pharmacogenetics of prednisone and prednisolone in patients with nephrotic syndrome. Pediatr Nephrol.

[35] Hodson E (2003). The management of idiopathic nephrotic syndrome in children. Paediatr Drugs.

[36] Davison AM, Lambie AT, Verth AH, Cash JD (1974). Salt-poor human albumin in management of nephrotic syndrome. Br Med J.

[37] Haws RM, Baum M (1993). Efficacy of albumin and diuretic therapy in children with nephrotic syndrome. Pediatrics.

[38] Serramontmany E, Muñoz M, Fernández-Polo A, Morillo M, Gómez-Ganda L, Cañete-Ramírez C, Ariceta G (2020). Home albumin infusion therapy, another alternative treatment in patients with congenital nephrotic syndrome of the Finnish type. Front Pediatr.

[39] Meena J, Bagga A (2020). Current perspectives in management of edema in nephrotic syndrome. Indian J Pediatr.

